# Unraveling the role of Breg cells in digestive tract cancer and infectious immunity

**DOI:** 10.3389/fimmu.2022.981847

**Published:** 2022-12-23

**Authors:** Helena Moreira, Agnieszka Dobosz, Łucja Cwynar-Zając, Paulina Nowak, Marek Czyżewski, Marta Barg, Paweł Reichert, Aleksandra Królikowska, Ewa Barg

**Affiliations:** ^1^ Department of Medical Sciences Foundation, Wroclaw Medical University, Wroclaw, Poland; ^2^ Faculty of Pharmacy, Wroclaw Medical University, Wroclaw, Poland; ^3^ Faculty of Medicine, Wroclaw Medical University, Wroclaw, Poland; ^4^ Department of Trauma Surgery, Clinical Department of Trauma and Hand Surgery, Faculty of Medicine, Wroclaw Medical University, Wroclaw, Poland; ^5^ Ergonomics and Biomedical Monitoring Laboratory, Department of Physiotherapy, Faculty of Health Sciences, Wroclaw Medical University, Wroclaw, Poland

**Keywords:** Breg cells, Bregs phonotypes, Bregs cytokines, microbiota, parasite diseases, cancers

## Abstract

Over the past two decades, regulatory B cells (Breg cells or Bregs) have emerged as an immunosuppressive subset of B lymphocytes playing a key role in inflammation, infection, allergy, transplantation, and cancer. However, the involvement of Bregs in various pathological conditions of the gastrointestinal tract is not fully understood and is the subject of much recent research. In this review, we aimed to summarize the current state of knowledge about the origin, phenotype, and suppressive mechanisms of Bregs. The relationship between the host gut microbiota and the function of Bregs in the context of the disturbance of mucosal immune homeostasis is also discussed. Moreover, we focused our attention on the role of Bregs in certain diseases and pathological conditions related to the digestive tract, especially *Helicobacter pylori* infection, parasitic diseases (leishmaniasis and schistosomiasis), and gastrointestinal neoplasms. Increasing evidence points to a relationship between the presence and number of Bregs and the severity and progression of these pathologies. As the number of cases is increasing year by year, also among young people, it is extremely important to understand the role of these cells in the digestive tract.

## 1 Regulatory B cells

B lymphocytes (B cells) are components of the immune system necessary for its proper functioning. Besides their best-known function, i.e. production of antibodies in contact with pathogens, B cells secrete immunoregulatory cytokines and act as antigen-presenting cells (APC) (1). Moreover, anti-inflammatory and immunosuppressive functions have been attributed to a subset of these cells, i.e. regulatory B cells (Bregs). The term “regulatory B cells” was used for the first time by Mizoguchi et al. in 2002 to describe the subpopulation of B cells that suppressed inflammatory bowel disease (IBD) (1, 2). Although evidence for a subset of B cells that may have immunosuppressive properties had emerged much earlier. For example, in 1974 Katz S.I. et al. noticed that after immunization of guinea pigs with ovalbumin in Freund’s incomplete adjuvant, B cells or their products suppressed the delayed-type hypersensitivity skin reactions by suppressing T lymphocytes (T cells) functions (3). Subsequently, the hypothesis has been confirmed in the early ninety eighties by Shimamura T. et al. The authors showed that splenic B cells, isolated after immunization of C57BL/6 mice with sheep erythrocytes (SRBC) and transferred to syngeneic mice, lead to suppression of immunoglobulin M (IgM) and immunoglobulin G (IgG) plaque-forming cells’ responses to SRBC. The mechanism of this immune B cell-mediated suppression was dependent on T-helper (Th) cells and different from that of antibody-mediated suppression (4). In the mid-1990s, Wolf S.D. et al. studied the influence of B cells on induction of CD4^+^ (cluster of differentiation 4) T cells mediated experimental autoimmune encephalomyelitis (EAE), using genetically B cell-deficient model mice susceptible to EAE. While these mice had a similar incidence rate of EAE induction compared to control mice, greater variability was observed on the day of onset, disease severity, and failure to fully recover. The authors suggested that B cells are not required for the activation of encephalitogenic T cells and induction of EAE but play a role in the immune regulation over the course of this disease (5). These, and other studies from the last decades, provided a new approach to B cell functions in regulating immune responses. Currently, it is known that Bregs regulate the inflammatory processes of a wide variety of diseases such as autoimmune diseases, allergies, viral infections, bacterial infections, parasitic infections, and tumors. In addition, Bregs are involved in some physiological processes such as pregnancy. The number of Bregs has been shown to change during pregnancy and remain low in non-pregnant women, suggesting that they may play a role as a critical regulator of immune status during pregnancy (6).

### 1.1 Origin of Bregs

Bregs have been detected in B cell subsets, mainly B1 and B2 B cells as well as plasmocytes (1). B1 cells primally develop from the fetal liver and later in life in peritoneal and pleural cavities. They are divided into two subsets, B-1a and B-1b. After their activation by cytokines or bacteria, they migrate and produce immunoglobulin A (IgA) and IgM antibodies in the omentum, lymph, spleen, and intestinal lamina propria. B2 cells are produced in bone marrow in adults and differentiate into follicular B or marginal zone B cells (7, 8). Less than 1% of circulating B cells show the Breg phenotype, but this population can increase in chronic inflammatory and autoimmune diseases, as well as after organ transplantation and infections (9, 10).

According to studies, two models of Bregs development have been noted. The first is analogous to the thymus-derived regulatory T cells (Tregs). In this model, a particular subset of B cells expresses a specific transcription factor that controls the expression of genes that induce the suppressive function of Bregs. Currently, this specific transcription factor, analog to forkhead box P3 (FoxP3) in Tregs, is unknown (11). However, Si Yu Yang et al. have suggested that potential candidates in the mice model include Sox5 (SRY (Sex determining region Y) - box transcription factor 5), Myc gene, and Atf3 (Activating Transcription Factor 3) (12). In the second model, it is assumed that B cells, in response to stimuli, can adopt regulatory phenotypes to suppress inflammation (11). Other well-known models include single-lineage and multi-lineage Bregs. The single-lineage model determines subsets of Bregs originating from the same progenitor cells. The second feature is an expression of single transcription factors. In this model, Bregs possess suppressive properties in all stages of differentiation. In the multi-lineage development of Bregs, each subset arises from other progenitor cells (13). In addition, it was proposed that different Bregs populations might originate from existing subsets of B cells when activation processes take place. In this model all activated B cells can transform into immunosuppressive Bregs. However, it is still an open question whether Bregs represent progenitor derived B cells subsets or came from B cells that acquire suppressive functions through a process involving TLRs and BCR/CD40 (11).

### 1.2 Phenotypes of Bregs

Bregs don’t have a specific unequivocal surface marker and don’t secrete soluble mediators that are unique to them. Bregs subsets include many phenotypically different cells, each secreting various substances with suppressive effects, e.g. interleukin 10 (IL-10), interleukin 35(IL-35), transforming growth factor beta (TGF-β) (6, 14). Heterogenity of Breg cells is associated with many cases, e.g., cellular plasticity, epigenetic regulations, and functional adaptation. Moreover Breg regulation is different in disease conditions than normal. Bregs cannot be differentiated from progenitor cells, they mainly develop from various subsets of B cells. Well-known B cell examples include T2 B cells, MZ (marginal zone) B cells, B1b cells, FO (follicular) B cells. Additionally, microenvironmental stimuli play an essential role in this process (also described in the next paragraph). In response to stimuli, B cells can differentiate into Bregs (15). In healths, the Bregs stay at stable, low level, but under pathologic conditions their number rises (16). Hence, it is proposed to describe Bregs by their ability to suppress the immune system (17). For example, B10 cells with the phenotype CD24^hi^CD27^+^ secrete IL-10, thereby regulating the secretion of TNF-α (tumor necrosis factor-alpha) by monocytes (18). Several reports indicate the role of IL-10-producing Bregs in transplantation. For example, the protective capacity of Bregs in prolonging allograft survival was demonstrated in a mouse model, and increased frequencies of circulating Bregs were found in human transplant recipients. However, major studies to characterize IL-10^+^ Bregs have been conducted in the context of autoimmunity; their relevance has been found in several autoimmune diseases such as systemic lupus erythematosus (SLE), rheumatoid arthritis (RA), progressive systemic sclerosis (PSS), atrioventricular septal defect (AVSD), and Marinesco-Sjögren syndrome (MSS) (19). IL-10 is produced by Bregs having different phenotypes, e.g. CD19^+^CD27^int^CD38^+^. Plasmablasts with CD19^+^CD27^int^CD38^+^ phenotype suppress the activity of dendritic cells (DCs) to generate autoreactive T cells (18). The same CD19^+^CD24^hi^CD38^hi^ phenotype occurs on transitional B cells, which inhibit the differentiation of naïve T cells into Th1 (T helper 1) and Th17 (T helper 17), and activate Tregs (19). IgA^+^CD138^+^PD-L1 plasmablasts secrete TGF-β to suppress cytotoxic T cells (18). CD1d molecule is expressed on the surface of both Bregs and invariant natural killer (iNKT). Bregs expressing CD1d play a role in the regulation of the immune response in inflammatory reactions and autoimmune responses *via* presenting lipids to iNKT and modulating antigen-specific immune responses (20). Many other human Bregs phenotypes have been found, including: CD19^+^CD25^hi^CD71^hi^, CD19^+^CD9^+^, CD19^+^CD5^+^CD1d^hi^, CD19^+^TIM-1^+^, CD19^+^CD5^+^IgM^hi^CD23^-^CD21T^-^CD11b^+^, IL-10^+^CD19^+^CD21^hi^CD24^hi^, IgA^+^CD138^+^PD-L1^−^IL-10^+^. Different Bregs phenotypes have been reviewed in the literature (6, 18, 19).

### 1.3 Cytokine-dependent suppressing mechanisms Bregs

**Figure 1:**
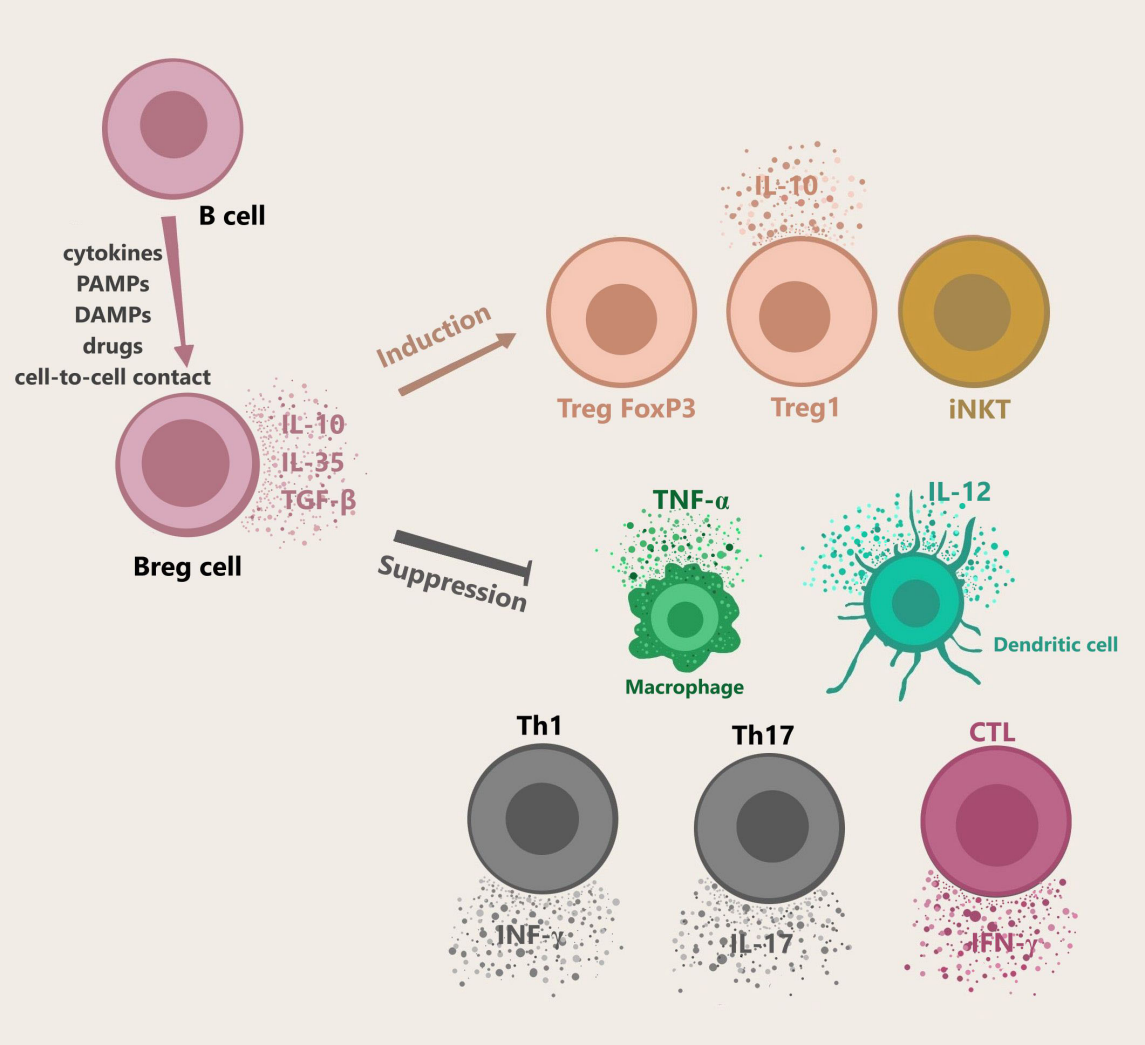
Bregs generation, cytokines secretion, and functions. B cells differentiate into Bregs by multiple mechanisms including cell-cell contact, cytokines, or other stimuli e.g. LPS. pDC drives the production of IL-10 secreting Bregs *via* IFN-α or CD40. Bregs release immunosuppressive cytokines like IL-10, IL-35, TGF-β. Granzyme B-expressing Bregs play a role in suppressing T cell responses. Activated Bregs induce naïve T cells to differentiate into Treg FoxP3 and Treg1, and take part in the maintenance of iNKT function. Moreover, Bregs participate the in suppression of Th1, Th17, CTLs, macrophages, and dendritic cells. *Abbreviations: Bregs, regulatory B cells; LPS, lipopolysaccharide; pDC, plasmacytoid dendritic cells; iNKT, invariant natural killer; Th1, T helper 1; Th17, T helper 17; CTLs*, cytotoxic T cells.

The primary function of Bregs includes maintaining the homeostasis of the immune system in case of infections, inflammation, or tissue damage. Bregs are activated by multiple mechanisms: cytokines (interleukin 1β, interleukin 2, interleukin 6, and interferon α), cell-to-cell interaction (e.g. CD40-dependent interaction with T cells), or various environmental stimuli such as lipopolysaccharide (LPS). Plasmacytoid dendritic cells (pDC) by delivering IFN-α and CD40-mediated stimulation also induce the production of IL-10 by Bregs (18). Bregs regulate the response of the immune system by secretion of cytokines, mainly IL-10, as well as IL-35 and TGF-β (1). These cytokines suppress the stimulation of macrophages and dendritic cells, CD4^+^ T cells, programmed death-1 T cells (PD-1^+^ T), and cytotoxic T cells (CTLs) (21, 22). An alternative way of downregulating the immune system by Bregs is the production of granzyme B, which suppresses CD4^+^ T cell proliferation. Although the exact mechanism of CD4^+^ T cells regulation by granzyme B is yet to be examined, it was proven to be contact-dependent and one of its mechanisms includes degradation of the ζ-chain of T Cell Receptor (TCR) (23, 24). Furthermore, it was shown that it is not dependent on competing for nutrients, PD-1 signaling pathway, or Fas/FasL pathway (**Figure 1**) (24, 25). Bregs activating factors were classified and are presented in **Table 1** (26–30).

**Table 1:** Bregs activating factors (26–30).

Breg activating factors	Examples
Cytokines	Interleukin-1β, interleukin-2, interleukin-6, interferon-α, GM-CSF
PAMPs	Bacterial PAMPs: H. felis, S. typhimurium, CpG ODN, LPS, M. tuberculosis
	Parasitic PAMPs: H. polygyrus, S. mansoni, L. major, B. malayi, parasitic egg-derived compounds like SEA, LNFPIII
	Viral PAMPs: RSV, HBV, HCV
DAMPs	DNA, RNA, fibrinogen, apoptotic cel, HMGB1
Drugs	Estrogen, Interferon-β, Glatiramer acetate, Tocilizumab
Cell-to-cell contact	T cells, pDC

CpG ODN, CpG oligodeoxynucleotides.

LNFPIII, lacto-N-fucopentaose-III.

SEA, soluble egg antigens.

pDC, plasmacytoid dendritic cells.

RSV, respiratory syncytial viruses.

GM-CSF, granulocyte-macrophage colony-stimulating factor.

IL-10 is a cytokine that can be produced by different subtypes of white blood cells, including T cells, B cells, natural killer (NK), macrophages, and dendritic cells. It can have both suppressive and stimulatory immunological effects (31). Some reports indicate that Bregs play a role in the differentiation of Tregs and induce FoxP3^+^ Tregs by secreting IL-10 (21, 22). Chimeric mice with Bregs deficiency show a decrease in Tregs and an increase in Th1 and Th17 which are responsible for inflammation (11, 22). A similar effect has been demonstrated on dendritic cells that differentiate from immature cells into tolerogenic cells under the influence of IL-10-secreting Bregs (10).

TGF-β^+^ Bregs play role in the differentiation of naïve T cells into Tregs. Natarajan P. et al. demonstrated that TGF-β caused inhibition of allergic reactions development in the respiratory tract using a two-phase mouse model of ovalbumin-induced asthma. TGF-β induced accumulation of FoxP3^+^ Tregs in the respiratory system and repressed eosinophils (32).

In contrast, less is known about the effects of IL-35 secreted by Bregs. IL-35 can covert B cells to Bregs and promote the expansion of Bregs and Tregs (10, 33). Simultaneously, IL-35 suppresses various members of the T cells family such as effector T cells, Th1, Th17, and other cells which take part in the immune response like macrophages (34). Studies have shown that IL-35 inhibits the immune response by regulating T cells. Mice whose B cells didn’t secrete IL-35 lost their ability to recover from EAE. In contrast to EAE, the lack of IL-35 in cell mice caused a rise in the immune response to *Salmonella typhimurium* infection due to increased macrophage and T-cell activation (35). Zhang et al. demonstrated that patients with ankylosing spondylitis had a lower level of Bregs compared to the control group. Moreover, the levels of IL-10 and IL-35 were also significantly lower in patients (36).

Recent studies show that the ratio IL-10/TNF-α is a better indicator of the regulatory function of Bregs than the level of IL-10 alone. Therefore, the cytokine profiles should be assessed rather than the level of individual cytokines (37, 38).

### 1.4 Cytokine independent suppressing mechanisms of Bregs

Bregs can also regulate immunological response in independent mechanisms without cytokines. These mechanisms include dependent on Bregs expression of glucocorticoid-induced TNF-α-related receptor ligand (GITRL), programmed death-1 ligand (PD-L1), and Fas Ligand (FasL) (1, 39). These molecules are essential to suppressive cytokine-independent mechanisms *via* inducing cell-to-cell contact. General functions include induction of Tregs, suppressions effector properties of T cells, and apoptosis of target cells (15). GITRL is involved in the control of Tregs proliferation. These molecules are expressed on APC like dendritic cells, macrophages, B cells as well as endothelial cells (40). Binding between GITRL on the Bregs surface and GITR receptors on T cells is observed in this process. Afterward, it induces proliferation and results in an increased level of CD4^+^ regulatory and effector T cells, but not CD8^+^ T cells (41). The GITRL is up-regulated by pro-inflammatory stimuli, while GITR can be up-regulated after T cells activation and tumor microenvironment factors (42). Blocking of GITRL on Bregs, in the EAE model, results in a low capacity to keep Tregs in the peripheral circulation and hinders EAE recovery. Moreover, high expression of GITR (Glucocorticoid-induced tumor necrosis factor receptor-related protein) on Tregs allows the binding of fc-GITRL (GITR ligand) and induces Tregs expansion (39, 43). Mechanism of action PD-L1 and FasL is similar. Molecules, which are expressed on surface of Bregs are bound with receptors on surface of T cells, like PD-1 and Fas. It induces apoptosis of T cells. IL-10 and IL-4 secretion by Bregs was also observed in this process (16). PD-L1 up-regulation is dependent on proinflammatory stimuli or dysregulation of signal transduction in tumor cells (44). Recently, it was discovered that PD-L1 is differentially expressed in B cells and that PD-L1^hi^ B cells inhibit follicular Th cells, leading to EAE suppression *via* regulation of humoral response. Moreover, Tregs were not required to induce a PD-L1^hi^ B cells mediated suppression suggesting their direct interaction with target T cells (45). On the other hand, in EAE, B cells *via* PD-L1 provided activating signals to Tregs resulting in the suppression of immune responses. Bregs have also been found to up-regulate PD-1 (programmed death receptor 1) on Tregs (39).

The immunomodulatory function of FasL was described by Tian J. et al. (42, 46). Transfer of LPS- activated B cells, expressing FasL, to non-obese diabetic (NOD) mice prevents the onset of autoimmune diabetes. It was associated with the secretion of TGF-β by B cells, inhibiting APC function and apoptosis on autoreactive T cells through the FasL (39, 46).

## 2 Microbiota and Bregs

### 2.1 Gut microbiota

The diversity of human gut microbiota has been an object of interest to scientists in biology and medicine for decades. Yet, we don’t fully understand the mechanisms between gut microbiota and their host - humans. In humans’ gastrointestinal tract live over 10^14^ microbes (47). A few factors play a huge role in the formation and development of human microbiota like genetic factors, environment, eating habits, and early exposure (48, 49). Bacterial infections, antibiotics, bad eating habits, and rapid changes in diet can all provoke dysbiosis of gut microbiota (50). Homeostasis between microorganisms and hosts is held through mucus, antimicrobial peptides, and IgA antibodies (51). Gut microbiota can interact with the brain through metabolites like short-chain fatty acids and subcellular elements of bacteria (52). Microbiota can be analyzed with 16S rRNA (ribosomal ribonucleic acid) sequencing. 16S rRNA contains 19 regions, 9 of them are variable, and 10 are stable among different bacteria strains, it is not a detailed analysis, but it is easy to perform and relatively not expensive. Sequencing variable 16S regions enables to identify specific strains in the microbiota (47, 53). A more complex analysis is Metagenomics, which is examining whole genetic material in a sample. The advantages of metagenomics are predicting the function of sequenced genes and providing a deeper analysis of differences between samples (47, 54). Another method is investigating metabolites exerted by microorganisms, which is called Metabolomics. Focusing on metabolites avoids the problem of inactive or unknown genes and lets us investigate the function of microbiota (47, 55). Lastly, it is possible to analyze the proteins of microbiota, this examination is called metaproteomics (47). To investigate the influence of microbiota it is common to examine germ-free (GF) mice, which are raised in sterile conditions or have undergone aggressive antibiotic therapy (56). GF mice let us investigate how the microbiota affects the host, manipulate the composition of the microbiota, settle only one species of bacteria into the host gut, and research its impact on the host, and with a genetic engineering, it is possible to examine the impact of the host on the microbiota. Unfortunately, the mice model rarely has a simple translation into humans (57). Lack of microbiota is not neutral for the host, Grover et al. showed that germ-free mice had larger colons and more liquid colon content, due to sodium and chloride secretion changes, compared to conventionally raised mice (58). Microbiota caused prolonged skin graft survival in mice compared to GF mice, the effect was linked to the presence of microbiota and IL-10-producing B cells. What is more, B cells of mice treated with antibiotics had a lower ability to produce IL-10 and suppress CD4^+^ T cells (37).

### 2.2 Interactions between microbiota and Bregs

How microbiota affects Bregs’ function are subjects of today’s research. Known molecules associated with bacteria interacting with Bregs are:

* Short Chain Fatty Acids (SCFA) - including butyrate, propionate, and acetate. They are metabolites of microbiota obtained by fermentation of polysaccharides undigested by host enzymes e. g. dietary fiber and resistant starch (59). It was shown that butyrate supplementation promotes the growth of bacteria, which metabolize tryptophan, through serotonin, to 5-hydroxyindole-3-acetic acid (5-HIAA). 5-HIAA activates the aryl-hydrocarbon receptor (AhR), which promotes the differentiation of spleen B cells into Bregs and it inhibits the differentiation of germinal center B cells and plasmablasts (60). What is more, the SCFA profile in stool and microbiota composition changed after induction of arthritis in mice, and it did not return to the pre-disease state in the remission phase. Changes included a lower level of butyrate in stool and a lower concentration of bacteria involved in SCFA production (60). This discovery may be important in terms of new methods of arthritis treatment (60, 61).
* Pathogen-associated molecular patterns (PAMPs)- which are molecules recognized by Pattern Recognition Receptors, that interaction leads to the activation of the immune system (57). In the case of Bregs, it seems the most important PAMPs are those identified by Toll-Like Receptors (TLR). Mishima et al. showed that bacterial TLR ligands like LPS or Pam3csk4 and lysate from colon microbiota bacteria *Escherichia coli (E. coli)* LF82, *Clostridium* species, *Enterococcus faecalis* promote IL-10 producing Bregs through TLR2 and MyD88 (myeloid differentiation primary response 88) protein (62).
* Bacteria’s DNA – which is also considered PAMP, may have a more significant clinical impact. In mice, the lupus erythematosus model after vancomycin administration, treatment with gut microbiota DNA solution, administered orally, promoted IL-10 and Bregs population. Compared to the control with vancomycin administration, the Breg population was bigger, the IL-10 level was higher, and the onset of lupus erythematosus appeared later (63). Bregs’ promotion by bacteria’s DNA is transmitted by TLR9 (64).

The impact of certain strains of bacteria is not the same in terms of the Bregs population. Maerz et al. showed that *Escherichia coli* promotes CD19^+^CD5^+^CD1d^+^IL10^+^ Bregs and CD19^+^TIM-1^+^ Bregs population and IL-10 production in greater quantities than *Bacteroides vulgatus* (65). This mechanism serves as a homeostatic loop, in which certain bacteria strains can live in the host gut and not be attacked by the host`s immune system. In the interaction, TLR2 and TLR4 seem necessary. What is worth noting, is the presence of *E. coli* downregulated MHC II (major histocompatibility complex II) on dendric cells, this process was promoted by IL-10 secreting Bregs. Further research needs to answer the question, of whether we can use highly immunogenic strains like *E. coli* in the regulation of autoimmune disease with Bregs anti-inflammatory function (65).

### 2.3 Bregs in H. pylori infection

Over than half of the world’s population is infected by *Helicobacter pylori* (*H. pylori*), and this gram-negative bacterium is considered a class 1 carcinogen by the World Health Organization (66). It is estimated that about 15% of people infected with *H. pylori* develop gastric ulcers (67). Epidemiological studies report that 2-3% of people infected with *H. pylori* eventually develop gastric cancer (68). Here we present reports showing the involvement of Bregs in *H. pylori* infection.

In the studies of Wei L. et al., a mouse model of *H. pylori* infection was used, and then the dynamic changes of IL-10-producing B cells and FoxP3^+^ Tregs in the gastrointestinal mucosa, spleen, and mesenteric lymph nodes after *H. pylori* infection were assessed. Based on the performed studies, it was observed that not only FoxP3^+^ Tregs but also IL-10-producing B cells can be expanded after *H. pylori* infection. Furthermore, it has been shown that IL-10-producing B cells can be induced 2 weeks after *H. pylori* infection in mice. Moreover, IL-10-producing B cells multiplied earlier than FoxP3^+^ Tregs. This suggests that IL-10-producing B cells may also play an important role in immunosuppression at an early stage of infection, before to induction of Tregs (68). Another experiment investigated the role of regulatory B lymphocytes in acute and chronic colitis following *H. pylori* infection. The group of mice infected with *H. pylori* showed a higher percentage of CD19^+^IL-10^+^ Bregs compared to the control group. The results of the research by Xia Li et al. suggest that CD19^+^IL-10^+^ Bregs may play a key role in the alleviation of acute and chronic colitis after *H. pylori* infection (69). Similar results were obtained in the study by Nahid-Samiei M et al., which compared the number of CD19^+^IL-10^+^ B cells in *H. pylori*-infected patients with cells in *H. pylori*-negative patients using the immunofluorescence method. Studies have shown that in patients infected with *H. pylori* the number of CD19^+^IL-10^+^ B cells was 2.5 times higher than in uninfected patients (P <0.0001). In addition, it was observed that the CD19^+^IL-10^+^ B cell count in infected patients with gastritis was 1.45 times higher than in infected patients with peptic ulcer disease. The authors of these studies claim that the increased number of CD19^+^IL-10^+^ B cells in *H. pylori*-infected patients and their association with other cells may play an important role in the pathogenesis of *H. pylori* infection (70). Chonwerawong M. et al. showed that abnormal NLRC5 (NOD-, LRR- and CARD-containing 5) signaling in macrophages may promote B cell lymphogenesis (CD19 ^+^) during chronic *H. pylori* infection. NLRC5 is an innate molecule of the immune system. NLRC5 is a negative regulator of gastritis and mucosal lymphoid formation in response to *H. pylori* infection (71). Zhang H. et al. demonstrated that in gastric cancer patients, follicular Th cells (Th1- Tfh cells) inhibited the formation of IL-10-secreting Bregs. Moreover, the frequency of Th1- Tfh cells was negatively correlated with the IL-10^+^ B cells in peripheral blood. This study showed that increased Th1-Tfh cell counts likely exacerbated tissue damage in *H. pylori*-infected individuals by suppressing the development of Bregs. The authors speculate that Bregs may play different roles at other stages of *H. pylori* infection and gastric cancer (72).

The above-described studies confirm that in *H. pylori* infection, an increased presence of Bregs is observed. Published studies showed that Bregs may play an important role in the pathogenesis of *H. pylori* infection and immunosuppression in the early stages of infection. It is assumed that Bregs may play different roles at different stages of *H. pylori* infection and gastric cancer, but more research is needed to elucidate their mechanism of action.

## 3 Bregs in parasite diseases

Helminthic infections are health problems but unfortunately in many cases, they are asymptomatic, which makes it difficult to diagnose them (73). Only long-term infections in children can give clinical symptoms such as retardation of growth, cognitive disturbances, or anemia (74). On the other hand, we have to remember that the stimulation of children by microbiome components such as bacteria, fungi, or parasites is necessary for the proper development of the immune system, but early exposition may also be dangerous or even life-threatening for neonates. Even in adults, parasite infection may cause gut microbiota distribution. However, it is known that parasitic worm infections cause a decrease in the level of autoimmune and allergic diseases in countries where parasitic worm infections are quite frequent (75). During evolution human host and nematodes have learned to coexist together thanks to special proteins produced by helminths. It helps them to manipulate the host’s immune response (76).

Helminths live in different environments such as the liver or intestine and for this purpose, they secrete different molecules that facilitate their staying inside the host by immunomodulation. One of the mechanisms is the production of tolerogenic Bregs which helps the parasites to survive (77).

It was proved some time ago that IL-10-producing B cells are induced during parasite infection. These stimulated B cells cause the human immune response that promotes parasite infection (20, 78). When Bregs were blocked the host resistance was increased and the parasite invasion was reduced (79). IL-10-producing B cells can also take part in the suppression of inflammation and the induction of immune tolerance. That was the reason why patients with autoimmune diseases were treated by live helminths (80).

**Figure 2:**
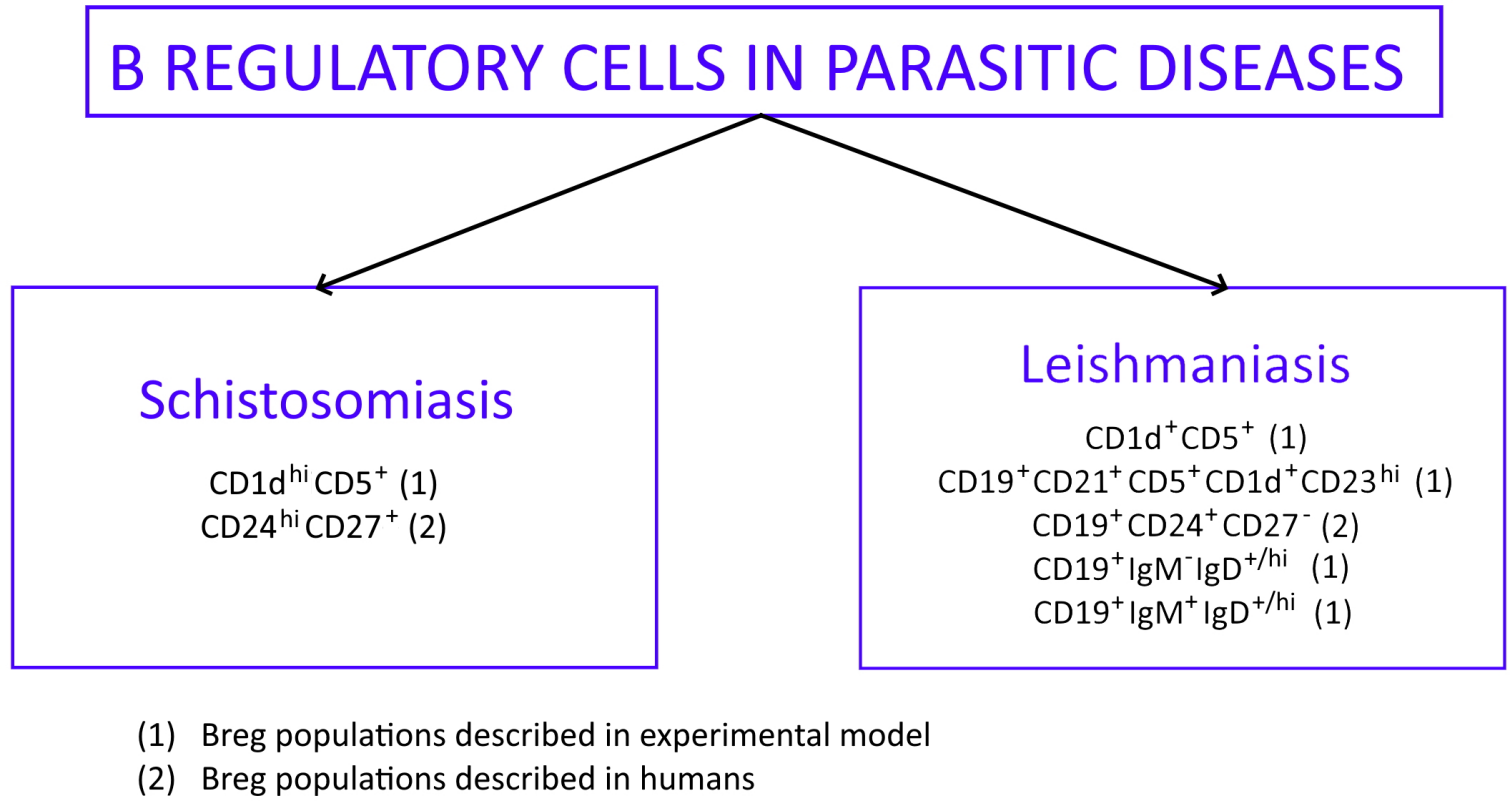
Phenotypic profile of Bregs described in parasitic diseases: schistosomiasis and leishmaniasis. Bregs phenotypes found in experimental models are marked (1) while Bregs phenotypes isolated from humans are marked (2).

There are two parasitic diseases connected with the gastrointestinal tract, where helminths cause immunological activation of Bregs: leishmaniasis and schistosomiasis (bilharzia). Bregs involved in these diseases express several surface markers based on the type of disease (**Figure 2**), but the transcription factor connected with the subset of B cells is still unknown (81). Some of them were isolated from humans during parasitic infections (marked with number 2 in **Figure 2**) (82, 83) and they produced large amounts of IL-10. The rest of the Bregs described in **Figure 2** were found only during *in vitro* experiments. They are marked with number 1 in **Figure 2** (21, 79, 84, 85).

### 3.1 Leishmaniasis

Leishmaniasis is a tropical parasitic disease caused by protozoa *Leishmania*. The picture of the disease varies from self-limiting cutaneous (CL) or mucocutaneous (MCL) forms to life-threatening organ forms called kala-azar and dum-dum fever. Infection of humans usually occurs as a result of mosquito bites or rubbing a squashed mosquito into a wound. It is also possible to transmit the parasite by blood transfusion, intravenous drug use, or vertically from mother to fetus. Inside the body, *Leishmania* infects macrophages which results in pathogen dissemination to the liver, spleen, and bone marrow (86).

Leishmaniasis is cutaneous in most cases but sometimes it can be visceral (VL) which is the most serious form of the disease. This form can be observed in Africa, Central and South Asia, Central America, and the Mediterranean basin in Europe. It leads to damage to internal organs and bone marrow. The incubation period of the disease is 3-6 months. The course of the disease can be fulminant; however, in most cases, the disease runs secretly for many months or years. Symptoms of the disease include increasing weakness and cachexia, prolonged, recurrent fever, diarrhea, and hyperpigmentation of the skin. Doctors may also find enlarged lymph nodes, liver, and spleen, fluid in the abdominal cavity, and edema. Patients are also predisposed to secondary bacterial infections of the lungs and gastrointestinal tract, tuberculosis, and septicemia. Coagulation disorders are the cause of life-threatening hemorrhages. Untreated visceral leishmaniasis leads to death in 95% of patients within two years from the onset of symptoms (85).

The involvement of B cells in the pathogenesis of leishmaniasis in humans is not entirely clear. Studies have shown that in experimental models of leishmaniasis, regulatory B cells play a negative role, contributing to increased susceptibility to infection by producing polyclonal antibodies and immunosuppressive cytokines (e.g. IL-10). High titers of leishmania-specific antibodies have been observed in patients with leishmaniasis, including type VL of the disease. B cells also appear to play a protective role in the pathogenesis of the disease, as manifested by the high prevalence in areas endemic to VL of healthy seropositive individuals. Also, after the infection is cured, antibodies to *Leishmania* persist for up to 15 years. Moreover, *in vitro* studies have shown that after contact with *L. infantum* amastigotes, B cells with a regulatory phenotype (CD19^+^CD24^+^CD27^-^) were produced, and they had a more remarkable ability to produce IL-10. Furthermore, when CD4^+^ T cells were contacted with a medium containing B cells incubated with *L. infantum* amastigotes, CD4^+^ T cell function, activation and proliferation were inhibited. This suggests that B cells possess regulatory activity mediated by IL-10 (81).

Using a similar methodology, B cells with a CD19^+^CD21^+^CD5^+^CD1d^+^CD23^hi^ phenotype associated with Bregs-mediated hypersensitivity were identified in mice by Bankoti et al. When these B cells were stimulated with *L. donovani* amastigotes, they produced large amounts of IL-10 (84).

In contrast, a study in dogs with visceral leishmaniasis indicated the presence of a novel post-type of IL-10-producing Bregs (CD19^+^IgM^-^IgD^+/hi^ or CD19^+^IgM^+^IgD^+/hi^). The level of these Bregs increased threefold or more during the development of leishmaniasis, and these cells suppressed Th1 cell effector functions through the interaction of B cells and PD-L1 (programmed death-ligand 1/receptor) (86). Very similar data were observed in a RA model where pathogenic B cells were present and expressed IgM together with IgD. Where IgM was expressed alone, no B cell pathogenicity was observed (87).

IL-10 is produced by macrophages, NK cells, dendritic cells, and many adaptive B and T cells. This cytokine, secreted by Bregs, influences the progressive weakening of the immune system in patients with VL, which may lead to death. Therefore, inhibition of IL-10 production or its neutralization (e.g., by blocking the IL-10 receptor or using anti-IL-10 monoclonal antibodies) would enable improved immune responses and parasite killing in both mouse and human VL infections. A complete understanding of the role of Bregs in leishmaniasis will help develop vaccines or immunotherapies and develop new strategies to regulate the function of these cells to gain the most benefit for VL sufferers (81).

### 3.2 Schistosomiasis (bilharzia)

Schistosomiasis is a parasitic disease that can have severe health consequences. It is the world’s most significant parasitological problem just after malaria. Hundreds of thousands of people die every year because of this disease. It is caused by a parasite worm *Schistosoma*. A person becomes infected when swimming in rivers or lakes. Cercaria (larval stage of *Schistosoma*) enters the human body through the skin. It localizes itself in the superficial blood vessels. Here it passes into the next stage - schistosomula - which, along with the blood, enters the lungs, the left side of the heart, the large bloodstream, and finally, the liver. Inside the liver, *Schistosoma* reaches its mature, sexual form. The male mates with the female, and together they enter the venous system of the pelvis minor, mainly the bladder. The parasites multiply in the venous vessels, and some of their eggs enter the lumen of the bladder or intestine and pass out of the human body with urine or feces. Whether the parasites and their eggs are located in the liver, bladder, or intestine (and sometimes in the lungs), they can cause different symptoms in their host. The first symptoms appear about 4-6 weeks after infection and are itching, erythema, and papular rash on the skin, and they disappear after 24-72 hours. In the next phase of the disease, associated with migration and reproduction of *Schistosoma*, general weakness of the body, sweats, chills, diarrhea (often bloody), hematuria, and weight loss can be seen. In the last phase, internal organs are damaged, especially the liver, spleen, ureter, and bladder. Advanced, chronic schistosomiasis also leads to intestinal polyposis with bloody diarrhea (88).

Bregs induced by *Schistosoma* infection can protect against allergic inflammation in mice and autoimmune diseases in humans. This was demonstrated by experiments in mice where it was found that B cells induced by chronic *Schistosoma* infection protected mice from airway allergic reactions (including anaphylaxis) in an IL-10-dependent manner. They were splenic CD1dhi IL-10-producing B cells with regulatory function and expressed CD5^+^, CD21^hi^, CD23^+^, and high levels of IgM. CD1d^hi^ cells can also be found in the peripheral blood of humans who are infected by *Schistosoma*. They produce significant amounts of IL-10 and can suppress T-cell proliferation *in vitro* (78).

In humans, autoimmunity inflammation, such as lupus, can be reduced when IL-10-producing B cells induce Tregs. This process can be promoted *via* schistosome-induced CD1d^hi^ B cells. Efforts were made to see the cellular mechanism that causes the development of Bregs. It was examined whether the regulatory potential of Bregs increases directly under the influence of solubilized *S. mansoni* egg antigens (SEA) or whether the effect on B cells is indirect through SEA-modulated macrophages. To solve this problem, mice were injected intraperitoneally with *S. mansoni* eggs or SEA alone, which significantly increased IL-10 expression by (3-fold) marginal zone B cells. Although both B cells and marginal zone macrophages bound SEA *in vivo*, macrophages appeared to be dispensable for Bregs induction. It was also found that one of the major SEA antigens, the secretory glycoprotein IPSE/alpha-1 (IL-4-inducing principle of *S. mansoni* eggs), also independently induced IL-10 production by naive B cells. Other schistosomal antigens such as kappa-5 and omega-1 had no such effect. The same effect of SEA and IPSE/alpha-1 was demonstrated in human CD1d^+^ B cells. In addition, SEA and IPSE-induced Bregs affected the development of Tregs *in vitro*. However, it is worth remembering that although Bregs induce Tregs, they have different roles in inflammation control (89).

Bregs suppress super inflammatory responses in allergic and autoimmune airway inflammation. Therefore, their induction may be used to treat several diseases. Helminths are particularly potent inducers of Bregs, so it is essential to understand the mechanisms of Bregs induction by helminths and identify the helminth-derived molecules involved in it. This may provide new opportunities for treating diseases with excessive inflammation (78, 90).

## 4 Bregs in cancers associated with the digestive tract

The development of immune tolerance is an essential factor responsible for cancer cells’ escape from immunosurveillance. A critical role in this phenomenon has been attributed to Tregs and immune-suppressive cytokines such as IL-10 or TGF-β. However, much evidence indicates that Bregs might also play a crucial part in the generation of cancer-immune tolerance and could serve as potential targets in cancer immunotherapy (91). Although the exact functions of Bregs in suppressing anti-tumor responses are still unclear, it can be speculated that Bregs function in two ways. First, they interact with other tumor-infiltrating immune cells, such as effector T cells, Tregs, myeloid-derived suppressor cells (MDSC), NK, and macrophages. Second, they interact directly with cancer cells (92). There are several pro-tumorigenic mechanisms by which Bregs can act. For example, by secretion of cytokines (TGF-β, IL-10, IL-35), they stimulated Foxp3 expression in Tregs and also suppressed CD4^+^ T cell proliferation in several tumors. Another mechanism implicated the interaction between PD-L1 and PD-1 expressed by Bregs and T cells, respectively. It was also found that IL-10-producing Bregs limit anti-tumor immunity *via* TNF-α and promote tumor development by attenuation of CD8^+^INF-ƴ^+^ T cells responses (93, 94).

IL-27 and IL-35-producing B cells (i27-Bregs and i35-Bregs) are B cells subclasses that are involved in cancer diseases. IL-27^+^ Bregs have been reported to be upregulated and associated with more aggressive diseases in patients with colorectal adenocarcinoma (CRC), liver metastases from CRC, hepatocellular carcinoma (HCC), and other solid tumors. The main mechanisms were through the increase of Tregs generation and inhibition of Th1/Th17 responses in gastric cancer (GC) or promoting T cell anergy in HCC (93).

i35-Bregs have been implicated as key players in the negative regulation of T cell immunity during cancer diseases (95). In pancreatic cancer, IL-35^+^ Bregs induced Treg proliferation and impair Th1/Th17 responses to enhance immune tolerance (9, 93).

In addition, in HCC, CD27^+^ expressing Bregs has also been reported to suppress T cell anti-tumor immunity and promote tumor progression through IL-10 secretion and PD-1 expression (95). In GC and CRC, CD27^+^ Bregs decrease INF-ƴ+, TNF, and IL-17 expression by T cells through IL-10. Also in HCC, CD27^+^ Bregs present in the tumor microenvironment cause the decrease and dysfunction of CD8^+^ T cells through IL-10 production, and their level is correlated with the disease stage and early recurrence (9).

**Figure 3:**
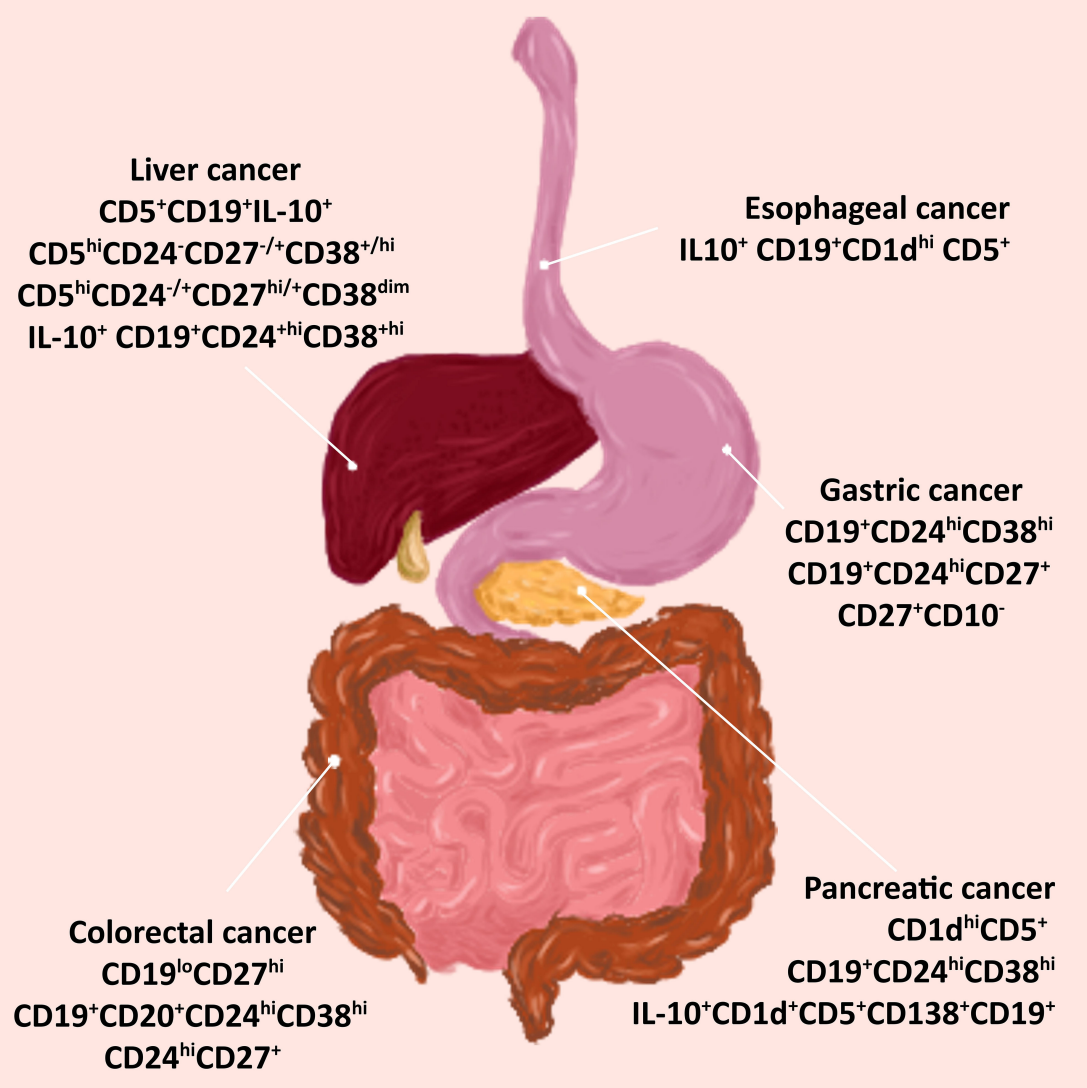
Bregs phenotypes in cancers associated with the digestive tract. Tumor-infiltrating Bregs were found in the most common types of cancer of the gastrointestinal tract. Various phenotypes have been detected in tumors of the esophagus, stomach, colon, pancreas, and liver. *Abbreviations: Bregs, regulatory B cells*.

The most common types of cancers of the gastrointestinal tract include esophageal cancer, gastric (stomach) cancer, colorectal cancer, pancreatic cancer, and hepatocellular (liver) cancer. Here we present reports that indicate the presence of tumor-infiltrating Bregs in the tumor microenvironment of these cancers and their relationship with disease progression. **Figure 3** shows different phenotypes of Bregs found in gastrointestinal cancers.

### 4.1 Esophageal cancer (EC)

Esophageal cancer is not widespread, although it ranks among the ten most common cancers worldwide. There are two main types of esophageal cancer: squamous cell carcinoma (ESCC) and adenocarcinoma. In patients with EC, increased frequency of Bregs (known as Br1 or B10) with IL10^+^ CD19^+^CD1d^hi^ CD5^+^ phenotype was associated with worsened clinical progression (96, 97). Qian L. et al. showed that the B10 ratio in the peripheral blood of EC patients increased significantly compared with the healthy control group. In addition, with higher clinical staging of EC, the immune function of patients was lower, and B10 expression was higher, suggesting that B10 may be related to the development of EC (98). Interestingly, EC-derived microvesicles (MVs) induce TGF-β^+^ Bregs with immune-suppressing activity toward CD8^+^ T cells (91). Mao, Y. et al. observed an elevated percentage of circulating B10 cells in ESCC patients compared with healthy controls. In addition, they demonstrated that exosomes from ESCC suppressed the proliferation of B cells and induced the accumulation of B10 and PD‐1^hi^ Bregs. The probable mechanisms of this phenomenon are the activation of TLR4 and MAPK (mitogen-activated protein kinase) signaling pathways by ESCC‐derived exosomes leading to PD‐1 expression and IL‐10 secretion in recipient B cells (96).

### 4.2 Gastric cancer (GC)

**Figure 4:**
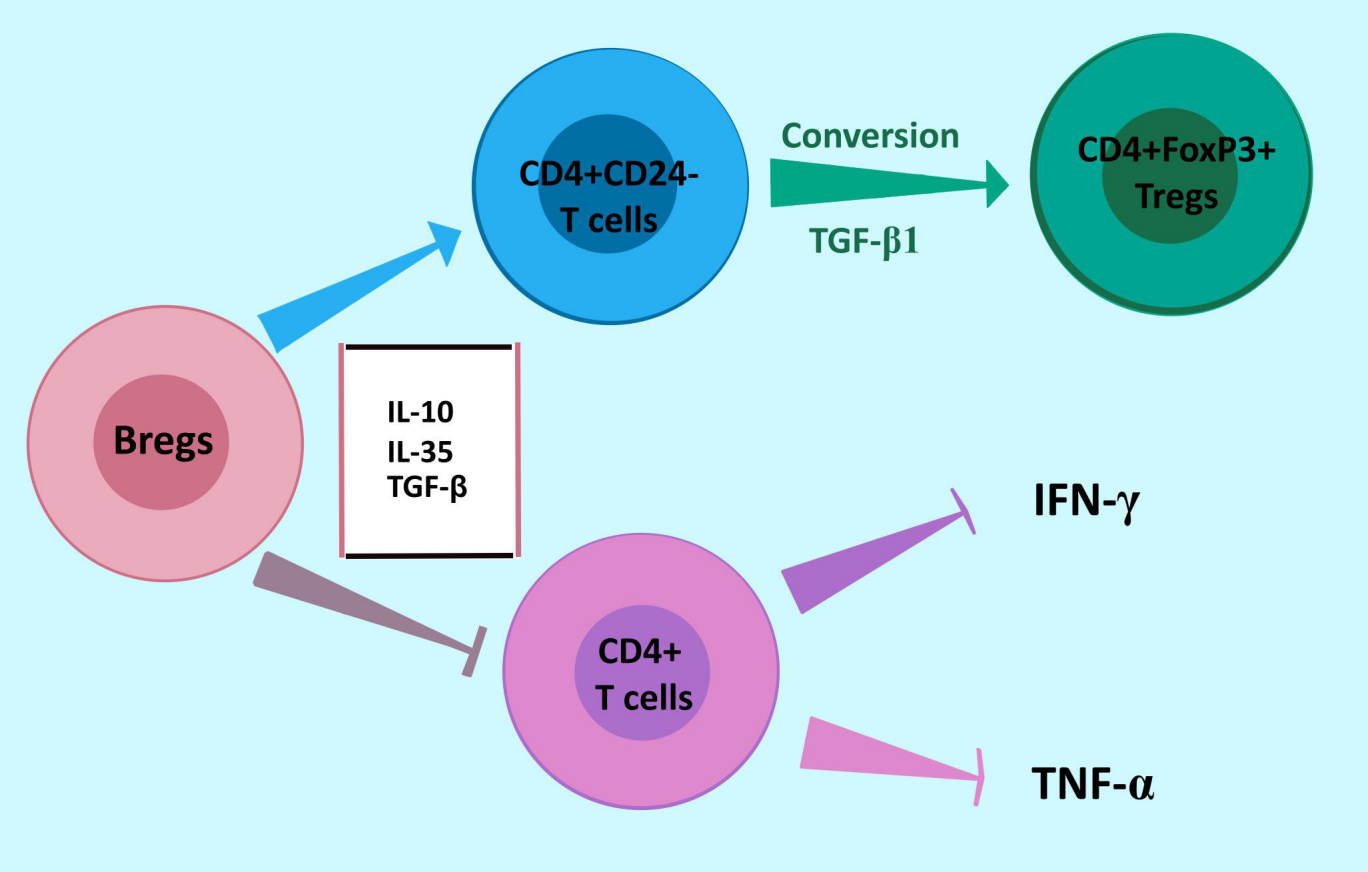
Bregs response in gastric cancer to promote immune escape. Bregs promote the conversion of CD4^+^CD25^-^ effector T cells to immunosuppressing CD4^+^FoxP3^+^ Tregs by producing TGF-β1. Moreover, Bregs impair the anti-tumor response by suppressing the secretion of IFN-γ and TNF-α by CD4^+^ T cells. *Abbreviations: Bregs, regulatory B cells*.

Gastric cancer cells are highly invasive and metastatic. It has been postulated that these properties are associated with the presence of immunosuppressive cells (also Bregs) secreting inhibitory cytokines in the tumor microenvironment (TME). In gastric cancer, the level of cells with inhibitory phenotype is significantly increased in the peripheral blood and TME. Accumulation of Bregs in GC may be linked to the presence of soluble factors secreted by tumor cells and present in TME (92, 97). Tumor-infiltrating Bregs in GC consist of three phenotypes: CD19^+^CD24^hi^CD38^hi^ transitional cells, CD19^+^CD24^hi^CD27^+^ memory cells, and IL-10 producing CD27^+^CD10^-^ cells (92, 99). Wang W. et al. demonstrated that CD19^+^CD24^hi^CD38^hi^ Bregs impair the anti-tumor response and promote GC immune escape in the TME by suppressing the secretion of IFN-γ and TNF-α by CD4^+^ T cells. CD4^+^ T cells are essential to the anti-tumor immune response. In addition, Bregs promote CD4^+^CD25^-^ effector T cells conversion into CD4^+^FoxP3^+^ Tregs by producing TGF-β1 in gastric cancer (**Figure 4**) (100, 101). Phenotype CD19^+^CD24^hi^CD27^+^ suppresses the production of INF-γ by CD4^+^ T cells and their proliferation. The frequency of Bregs in GC is positively correlated to the clinical stage and prognosis of patients (97). It was shown that XELOX chemotherapy (capecitabine plus oxaliplatin, first-line therapy for patients with metastatic gastrointestinal cancers) significantly decreases the number of CD19^+^CD24^hi^CD27^+^ cells in peripheral blood of GC patients and is associated with better prognosis (91). While in GC tissue, both CD27^+^CD10^-^ and CD27^-^CD10^-^ tumor-infiltrating B cells are present, only CD27^+^CD10^-^ phenotype produces IL-10 (100). Importantly, IL-10^+^ B cells in the tumor were increased in patients at more advanced tumor stages (102). Also, the frequency of IL-35-producing B cells is significantly upregulated in advanced GC. These findings indicate that the Bregs subset may participate in GC progression (103).

### 4.3 Colorectal cancer (CRC)

Inflammation (colitis) and chronic inflammatory diseases such as IBD play an important role in the initiation of colorectal cancers (CRC). Inflammatory T cells cytokines, such as IFN-γ and TNF-α are upregulated during the formation of aberrant crypt foci and colorectal polyps in an early stage of CRC and contribute to the spread of inflammation by increasing vascular permeability, production of other proinflammatory molecules, and promotion of effector T cell development. Mao H. et al. identified a specific subtype of regulatory B cells in TME of CRC, the CD19^lo^CD27^hi^ plasmablasts, which presented high IL-10 expression but not TGF-β. In addition, these plasmablasts showed lower CD24, CD38, and IgA expressions compared to the IL-10^−^CD19^lo^CD27^hi^ subset. However, the IL-10^+^CD19^lo^CD27^hi^ phenotype demonstrated potent activity in suppressing IFN-γ and TNF-α but did not promote FoxP3 expression. That indicates the role of these Bregs in CRC initiation (92, 104). Two other Bregs subsets were also found in CRC: CD19^+^CD20^+^CD24^hi^CD38^hi^ and CD24^hi^CD27^+^. The frequency of CD24^hi^CD27^+^ is significantly higher in CRC patients, however, did not correlate with tumor stage. Instead, the CD19^+^CD20^+^CD24^hi^CD38^hi^ phenotype increases in the advanced stage of the disease and metastatic tissue (92, 105). A study using microRNA (MIR15A and MIR16-1) knockout mice demonstrated an increased level of IgA^+^ B cells in colorectal tumor tissue. IgA^+^ B cells showed high expression of immune-suppressive cytokines IL-10 and TGF-β, as well as PD-L1; and were able to repress CD8^+^ T cells. Both MIR15A and MIR16-1 play a role in the regulation of cell proliferation, apoptosis, and drug resistance, and there was a negative correlation between the frequency of IgA^+^ B cells in CRC tissue and survival. These findings indicate that MIR15A and MIR16-1 might be a target for restraining immunosuppressive Breg cells in CRC (92, 106).

### 4.4 Hepatocellular carcinoma (HCC)

HCC is the most common type of primary liver cancer. However, the liver is also a frequent place of metastases, with induction of secondary/metastatic liver cancer. Bregs suppress antitumor immunity and promote liver cancer progression *via* several mechanisms in which IL-10, TGF-β, or IL-35 plays a crucial role. These mechanisms include CD40/CD40L signaling-mediated cytokine production, downregulation of TNF-α or Th17, upregulation of Treg, and transition of naive T cells into Tregs with subsequent suppression of the anticancer immune response (107, 108). Chen et al. found significantly higher expression of CD5^+^CD19^+^IL-10^+^ Bregs in the peripheral blood of HCC patients than in healthy controls (109). Recently, Ye L. et al. identified a protumorigenic TIM-1^+^ Bregs subset with CD5^hi^CD24^-^CD27^-/+^CD38^+/hi^ phenotype in the HCC microenvironment. TIM (T cell Ig and mucin domain) is a transmembrane glycoprotein with immunoregulatory function, present on Bregs. TIM-1^+^ Bregs create an immunosuppressive TME by secreting IL-10 and impairing CD8^+^ T cells, leading to inhibition of TNF-α and INF-γ secretion. Strong infiltration of TIM-1+ Bregs in TME is correlated with advanced disease stage and poor survival. In addition, HCC tumor-derived exosomes induced the transformation of B cells into TIM-1^+^ Bregs (29). Another protumorigenic subset of B cells, identified in advanced-stage HCC, constitutively expressed higher levels of PD-1. These Bregs exhibited a unique CD5^hi^CD24^-/+^CD27^hi/+^CD38^dim^ phenotype, different from conventional CD24^hi^CD38^hi^ peripheral Bregs. PD-1^+^ Bregs operate *via* IL-10-dependent pathways to induce CD8^+^ T cell dysfunction and thereby create conditions that are conducive to tumor progression (110). In post-hepatitis HCC patients, a higher frequency of peripheral IL-10^+^ CD19^+^CD24^+hi^CD38^+hi^ Bregs was found compared to patients with chronic HCV (Hepatitis C Virus), HCV-related liver cirrhosis, or healthy controls and was positively correlated with CD4^+^FoxP3^+^ Tregs and serum IL-10, IL-35 levels (111). Recently, it was stated that Bregs promote the growth and invasion of HCC by direct interaction with HCC cells through CD40/CD154 signaling. Furthermore, PD-1^hi^ B cells were described as the primary subtype of Bregs in human HCC operating through an IL-10-dependent pathway to induce T-cell dysfunction (112). Tumor-infiltrating B cells that express high levels of CD5^+^ have been shown to promote tumor angiogenesis/growth through lymphotoxin production and have been associated with poorer clinical outcomes in HCC (113).

### 4.5 Pancreatic cancer (PC)

PC is associated with a smaller mutational burden and more robust microenvironmental mechanisms of immune tolerance compared to other cancers such as colon (114). Zhao Y. et al. found that higher Bregs levels existed in the peripheral blood of PC patients than in healthy controls and were associated with worse overall survival. Moreover, the frequency of Bregs was higher in PC patients with invasion and/or metastasis and positively correlated with the tumor-node-metastasis (TNM) stage of PC. It was found that PC cell-derived IL-18 (interleukin 18) increases Bregs-induced immunosuppression. In patients with PC, the levels of IL-18 are significantly increased and have been associated with tumor progression and a worse prognosis. It was demonstrated that IL-18 promotes B cell proliferation upregulates IL-10 expression and PD-1 in B cells as well as inhibits the antibody-dependent cellular cytotoxicity of Tc cells and NK cells (115). Another study, using a mouse model of pancreatic intraepithelial neoplasia (PanIN), demonstrated the enrichment in IL-10 and IL-35 producing immunosuppressive CD1d^hi^CD5^+^ Breg cells in the PanIN microenvironment. The authors identified IL-35 as a prominent downstream effector of CD1d^hi^CD5^+^ Bregs function in pancreatic tumorigenesis by directly stimulating tumor cell proliferation (116).

PC progression was also associated with the upregulation of CD19^+^CD24^hi^CD38^hi^ Bregs phenotype. Furthermore, this Bregs subset was positively correlated with the frequency of CD4^+^CD25^hi^ Tregs suggesting that they impact tumor progression through Tregs (117). Shen L. et al. showed that IL-10^+^CD1d^+^CD5^+^CD138^+^CD19^+^ Bregs is an important factor in the modulation of immunosuppressive TME in various desmoplastic murine tumor models, including PC. They showed that inhibition of CXCL13 (C-X-C motif chemokine ligand 13), produced by tumor-associated fibroblasts present in TME suppresses PC tumor growth. CXCL13 is responsible for the recruitment of B cells to the TME, which then differentiate into IL-10^+^CD1d^+^CD5^+^CD138^+^CD19^+^ cells. This highlights the relationship of Bregs with PC tumor escape from immunosurveillance (118).

In summary, the general role of Bregs in cancer associated with digestive tracts is to impair anti-tumor immunity, which helps tumors escape from immunosurveillance. The frequency of Bregs is closely associated with clinicopathological factors. Immunotherapy targeting these cells has emerged as a therapeutic option that may contribute to disease remission and improve patient survival.

## 5 Conclusions

Despite the still relatively incomplete knowledge, it is now widely accepted that the normal immune system has, in addition to regulatory T cells, a population of B cells specialized in immune suppression. However, the lack of specific Bregs markers makes it difficult to characterize and study their functionality (11). So far, several phenotypes of Bregs have been recognized in various pathological conditions, emphasizing their diversity and role in the development of diseases on an inflammatory, infectious, and neoplastic basis (15). Increasing evidence points to a relationship between the presence and amount of Bregs with the severity and progression of the disease, which makes them an essential therapeutic target. This is extremely important for disorders related to the digestive tract and its associated organs due to the increasing frequency of the diseases, even in younger patients (119). Nowadays, diets, “chemical” food, and bacterial microflora in the gut are the most contributing factors to this phenomenon. More research is needed to address the various mechanisms involved in Breg’s role in these diseases. Many questions remain unanswered, e.g. whether Bregs dysfunction is directly correlated with the development and pathogenesis research, or whether they play an only immunoregulatory role. Based on such discoveries, developing a new therapeutic strategy will be possible.

## Author contributions

HM and AD took the lead in design and conception of the article and in writing the manuscript, wrote sections of the manuscript, wrote the manuscript with input from all authors. EB, and ŁC-Z contributed to conception and design, wrote sections of the manuscript. PN, MC, and MB wrote sections of the manuscript. PN prepared the figures. PR and AK contributed to the final version of the manuscript. All authors contributed to manuscript revision, read, and approved the submitted version.
